# Evaluation of complete response to azacitidine according to the revised International Working Group 2023 response criteria for higher risk MDS. Does it make a difference in patients’ outcome?

**DOI:** 10.1038/s41375-023-02051-3

**Published:** 2023-10-10

**Authors:** Anthi Bouchla, Sotirios G. Papageorgiou, Argyris Symeonidis, Ioanna Sakellari, Panagiotis Zikos, Thomas P. Thomopoulos, Eleftheria Hatzimichael, Athanasios Galanopoulos, Nora-Athina Vyniou, Ioannis Kotsianidis, Vasiliki Pappa

**Affiliations:** 1 Second Propaedeutic Department of Internal Medicine, National and Kapodistrian University of Athens, Athens, Greece; 2 Department of Internal Medicine, University Hospital of Patras, Rio, Greece; 3 Department of Hematology and Stem cell Transplantation, Georgios Papanicolaou General Hospital, Thessaloniki, Greece; 4 Department of Hematology, Aghios Andreas General Hospital, Patras, Greece; 5 Department of Hematology, University Hospital of Ioannina, Ioannina, Greece; 6 Department of Hematology, Laikon General Hospital, Athina, Greece; 7 Department of Hematology, Democritus University of Thrace Medical School, Alexandroupolis, Greece

**Keywords:** Risk factors, Myelodysplastic syndrome

**TO THE EDITOR:**

Higher-risk myelodysplastic syndromes (HR- MDS) are characterized by an increased risk of acute myeloid leukemia (AML) transformation and compromised overall survival (OS) [1, 2]. The only curative treatment is allogeneic hematopoietic stem cell transplantation (allo-HSCT); however, the vast majority of patients are ineligible for this treatment owing to advanced age. Therefore, for these patients, the treatment goal is to prevent disease transformation and prolong OS. In MDS clinical trials, standardized treatment response criteria are an absolute requirement for assessing and comparing the efficacy of new therapeutic agents [3], and they are often considered as surrogate markers for OS, as in the case of azacitidine in HR- MDS [4, 5]. In the AZA001 study, patients with HR-MDS who achieved CR, partial response or hematologic improvement had significantly longer OS, compared to those treated with conventional care regimens [6]. Moreover, our group has shown that the achievement of stable disease was associated with significantly longer OS [7]. Therefore, the significance of achieving CR after azacitidine treatment is not the only factor affecting prognosis.

According to the International Working Group (IWG) 2006 response criteria, complete response (CR) requires the presence of ≤5% marrow blasts, no detectable peripheral blood (PB) blasts as well as hemoglobin (Hb) ≥ 11 g/dL and platelets ≥100 × 10^9^/L. In the case of not recovered blood counts, marrow CR (mCR) is defined as ≤5% marrow blasts and decrease by ≥50% over pretreatment, while no PB response is required [8].

The latest consensus proposal for revised IWG2023 response criteria for HR- MDS introduces three major alterations in the previous IWG 2006 criteria, which are relevant to CR definition: First, marrow-blast threshold drops to <5%, second, Hb cut-off is lowered to 10 g/dL and third, mCR breaks into the provisional entities CR_L_ (CR_uni_ and CR_bi_- according to one or two of the following respectively: Hb ≥ 10 g/dL; platelets ≥100 × 10^9^/L; neutrophils ≥1.0 × 10^9^/L) and CR_h_ (no evidence of CR_L_ but both platelets ≥50 × 10^9^/L and neutrophils ≥0.5 × 10^9^/L). Cases not fulfilling CR_L_ or CR_h_ criteria are not considered CR [9].

IWG MDS response classification systems have two drawbacks: First, they are based on consensus, and second, they are designed to determine drug efficacy in clinical trials. The question is whether they can be applied in real-life using currently approved treatments, such as azacitidine. Furthermore, it would be of interest to compare these two response classification systems regarding their ability to predict survival in a given patient population. To address these matters, we searched the Hellenic (Greek) MDS registry for azacitidine treated HR-MDS patients with excess blasts per WHO2016 criteria [10] (defined as ≥5% bone marrow blasts) at treatment initiation, who achieved CR or mCR by the IWG2006 criteria. In a total of 572 HR-MDS patients, 103 (18%) patients had documented CR or mCR, consistent with that previously reported in a pooled analysis of population-based studies [5]. All patients were registered between January 2010 and August 2018 with last follow-up in January 2019. The current study was performed in accordance with the ethical standards of the World Medical Association Declaration of Helsinki (version 2008) and written informed consent was obtained from all patients. The study was approved by the “Attikon” University Hospital Institutional Review Board (approval number: 17-04-2018 Γ.8).

Of these 103 CR/mCR patients, 95 were selected according to the IWG2023 definition of CR that excludes patients with 5% marrow blasts at reevaluation. CR was further defined using the IWG2006 and the IWG2023 criteria and patient population characteristics are shown in Table 1.

**Table 1:** Population characteristics and CR assessment according to the IWG2006 and the IWG2023 response criteria.

Total patients (*N*) with ≤ 5% blasts at reevaluation	103
Age (years)	76 (57–86)
Female/Male (*N*)	30/73
IPSS-R at treatment start (intermediate/high/very high (*N*)	25/43/35
IWG2006 response CR/mCR with HI/mCR without HI (*N*)	70/26/7
Median follow-up (months)	24 (6.5–67)
Number of AZA cycles at best response (*N*)	6 (3–14)
Number of Discontinued/allo-Transplanted/Ongoing patients at last follow-up	70/6/27
Total patients (*N*) with <5% blasts at reevaluation	95
IWG2006 response CR/mCR (*N*)	65/30
IWG2023 response CR/CR_bi_/CR_uni_/CR_h_/less than CR_h_ (*N*)	72/20/3/0/0
Redefined cases (*N*)	30
mCR to CR using the 10 g/dL threshold (*N*)	7/30
mCR to CR_bi_ (*N*)	20/30
mCR to CR_uni_ (*N*)	3/30

Continuous variables are expressed as median (5–95% range).

We subsequently investigated whether patients achieving mCR without hematological improvement (HI) according to the IWG2006 had a worse prognosis compared to those in mCR with HI. Cox proportional hazard analysis (HR (95% CI), *p* values) comparing patients in the above groups was as follows: OS: 2.194 (0.800–6.017), *p* = 0.127, EFS: 0.905 (0.379–2.158), *p* = 0.820, AML-FS: 1.893 (0.716–5.007), *p* = 0.219. Unfortunately, the small number of patients in mCR with or without HI did not allow us to draw reliable conclusions.

We compared OS, EFS, and AML-FS in our study population including patients with 5% blasts (103 patients) against the same population but keeping only patients with ≤5% blasts (95 patients). Kaplan–Meier curves were almost identical (data not shown), so we concluded that the lowering of blast threshold per the new IWG2023 criteria did not change the results in our study population. However, we need to take into account that our study population was relatively small and studies including more patients are needed, in order to clarify this issue.

Finally, we performed survival analysis using the Kaplan–Meier method and the log-rank test to compare survival probabilities and Cox- proportional hazards to determine Hazard Ratios with 95% Confidence Intervals (CI) between response categories as defined per IWG2006 or IWG2023 criteria in the selected 95 patients.

**Fig. 1:**
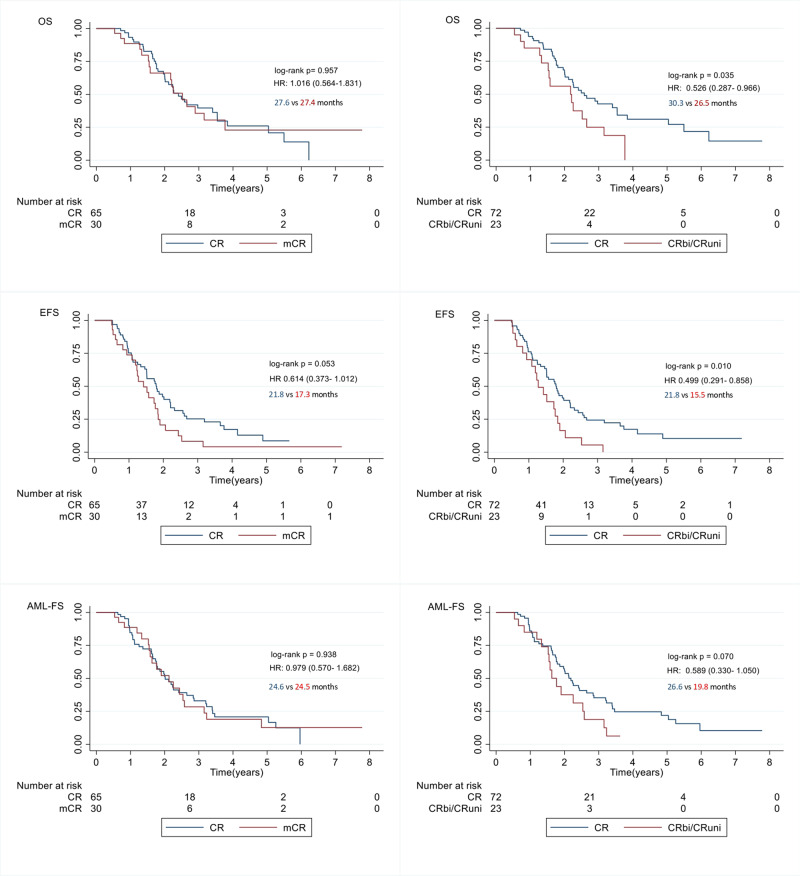
OS, EFS, AML-FS by the IWG2006 criteria (left column) and the IWG2023 criteria (right column). Time in months refers to median survival.

To perform survival analysis, patients in CR_bi_ and CR_uni_ were categorized as one group, since there were only 3 patients in the CR_uni_ group. All patients were censored upon allo-HSCT. For Event Free Survival (EFS), events were defined as relapse, or progressive disease (PD), or AML transformation or death, following this order. AML transformation was defined as increase in blasts ≥20%. AML-Free Survival (AML-FS) events were defined as AML transformation or death. Survival curves for OS, EFS, and AML-FS per IWG2006 and IWG2023 are depicted in Fig. 1.

As seen in the figure, the achievement of CR vs mCR or CR_uni_/_bi_ has a stronger impact on EFS than OS. A possible explanation is that EFS is directly dependent upon the response to treatment under study, while OS also depends on the response to following treatments after initial treatment failure, as previously suggested by Garcia et al. [5]. In their study, the authors showed a rather weak correlation between CR rate and OS in HR-MDS azacitidine treated patients in the real-world setting. By definition, EFS accounts for duration of response as well as response itself, which is of particular relevance regarding patient outcome.

More importantly, survival curves are more distinctively separated for CR vs CR_bi_/CR_uni_ (IWG2023) than for CR vs mCR (IWG2006); in addition, the log-rank test is more statistically significant respectively for all three categories: OS, EFS, and AML-FS. Therefore, it appears that reclassification of these patients from mCR to CR by the new IWG2023 criteria is valid. In fact, the results of our study show that lowering of the Hb cut-off value alone from 11 g/dL to 10 g/dL was enough to improve outcome prediction in azacitidine treated HR-MDS patients. Moreover, considering that residual normal hemopoiesis is diminished in MDS patients, the Hb cut-off value of 11 g/dL might have been too strict, and a lower cut-off value would be more relevant. Owing to the small number of patients, our study cannot answer if reclassification of mCR to CR_L_ and CR_h_ is of importance.

In our study, the percentage of patients who underwent allogeneic transplantation was relatively low (6/95 patients with <5% blasts upon reevaluation). Older age, comorbidities and operational challenges mainly accounted for this low rate in our study population.

In conclusion, we have shown that reclassification of CR with the updated IWG2023 criteria can better predict outcome compared to the formerly used IWG2006 criteria in a retrospective cohort of HR-MDS patients treated with azacitidine. In our cohort, this improvement is attributed exclusively to the new proposed Hb cut-off value. More studies are required to validate the prognostic significance of the IWG2023 compared to the IWG2006 criteria in this patient population.
